# Supplementary material for: Unraveling the role of the mitochondrial one-carbon pathway in undifferentiated thyroid cancer by multi-omics analyses
Source: Nat Commun. 2024 Feb 8;15:1163. doi: 10.1038/s41467-024-45366-0 (PMC10853200; doi:10.1038/s41467-024-45366-0)
Supplement: Supplementary file 1 — Supplemental information [file 41467_2024_45366_MOESM1_ESM.pdf]

Unraveling the role of the mitochondrial one-carbon pathway in undifferentiated thyroid cancer by multi-omics analyses

Seong Eun Lee, Seongyeol Park *et al.*

Supplementary information

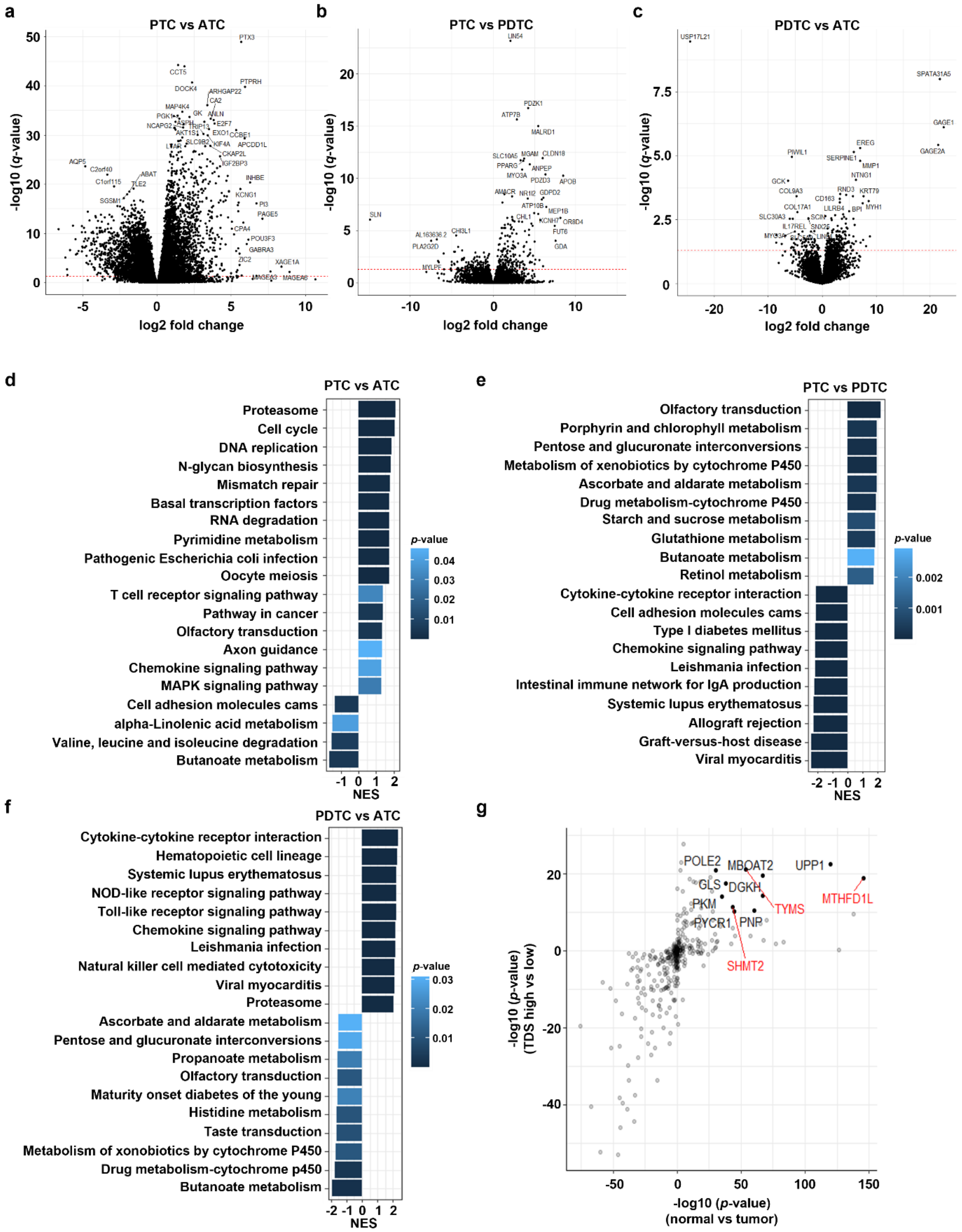

Supplementary Figure 1. Comparison of SGP genes among thyroid cancer types using bulk RNA sequencing data from our cohort. a-c Volcano plot comparing gene expression among cancer types: PTC (n=348) versus ATC (n=16) (**a**), PTC (n=348) versus PDTC (n=5) (**b**), and PDTC (n=5) versus ATC (n=16) (**c**). **d-f** Differentially enriched KEGG pathways from GSEA among cancer types: PTC (n=348) versus ATC (n=16) (**d**), PTC (n=348) versus PDTC (n=5) (**e**), and PDTC (n=5) versus ATC (n=16) (**f**). **g** Scatter plot showing DEGs between tumor versus normal (horizontal axis) and TDS-low versus TDS-high tumors (vertical axis), simultaneously. *P*-value of differential expressed genes (DEG) and gene sets enriched analysis

(GSEA) was performed using the DESeq2 and fgsea R package, respectively. PTC, papillary thyroid cancer; ATC, anaplastic thyroid cancer; PDTC, poorly differentiated thyroid cancer; NES, normalized enrichment score; TDS, thyroid differentiation score. Source data are provided as a Source Data file.

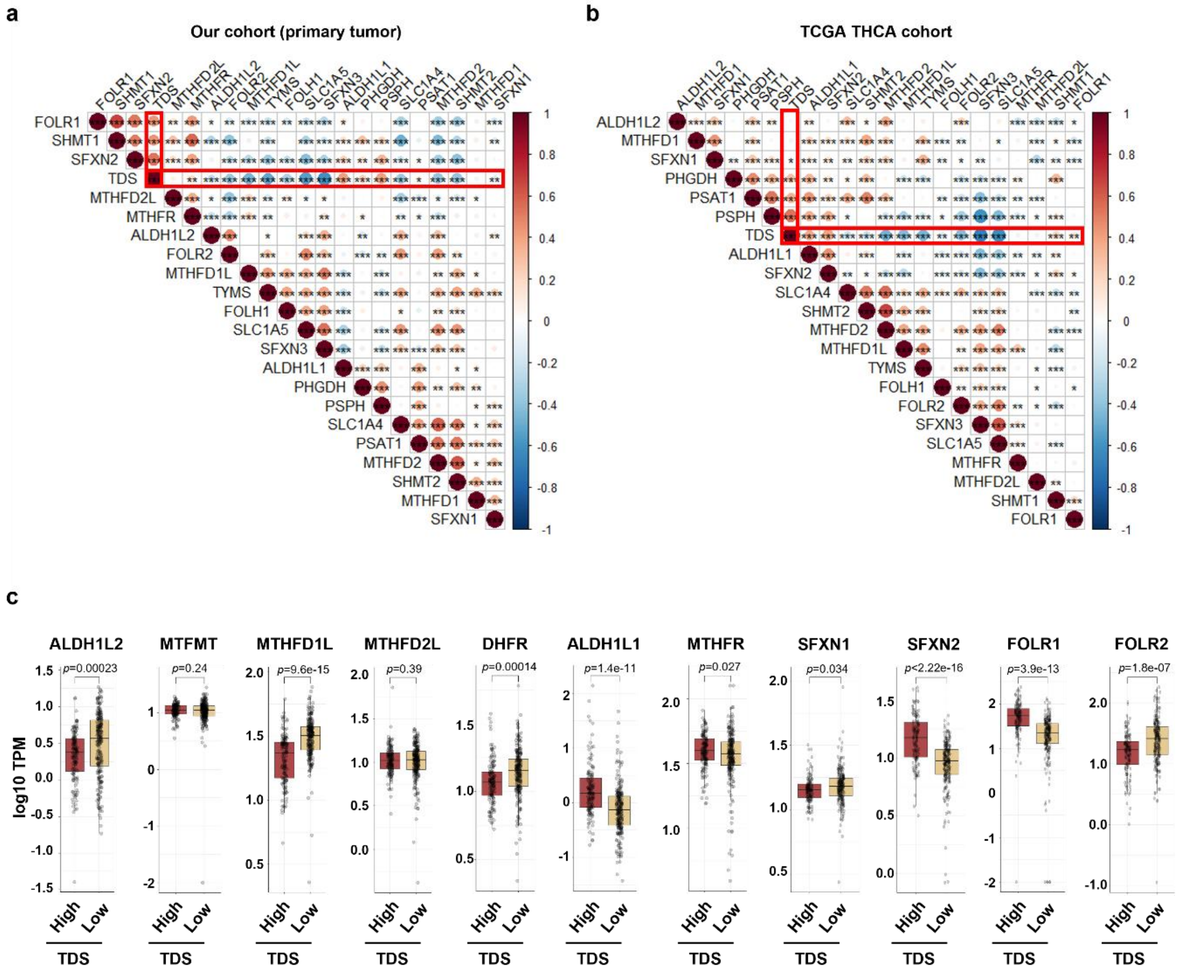

Supplementary Figure 2. Association between the SGP and TDS using bulk RNA sequencing data. a, b Correlation plots between thyroid differentiation score (TDS) and serine/glycine metabolic pathway (SGP) genes in primary tumors from our cohort (**a**, n=369) and from the TCGA cohort (**b**, n=500). The color indicates Pearson's correlation coefficients. The R package "corrplot" was used. **c** Box plots comparing expression of SGP genes between TDS-high and -low groups. TPM, transcripts per million. Data were expressed as the mean ± SD. A student's *t* test (two-sided) was used for statistical analysis. *, p <0.05; **, p <0.01; ***, p <0.001. Exact p values shown in a-b can be found in source data file. Source data are provided as a Source Data file.

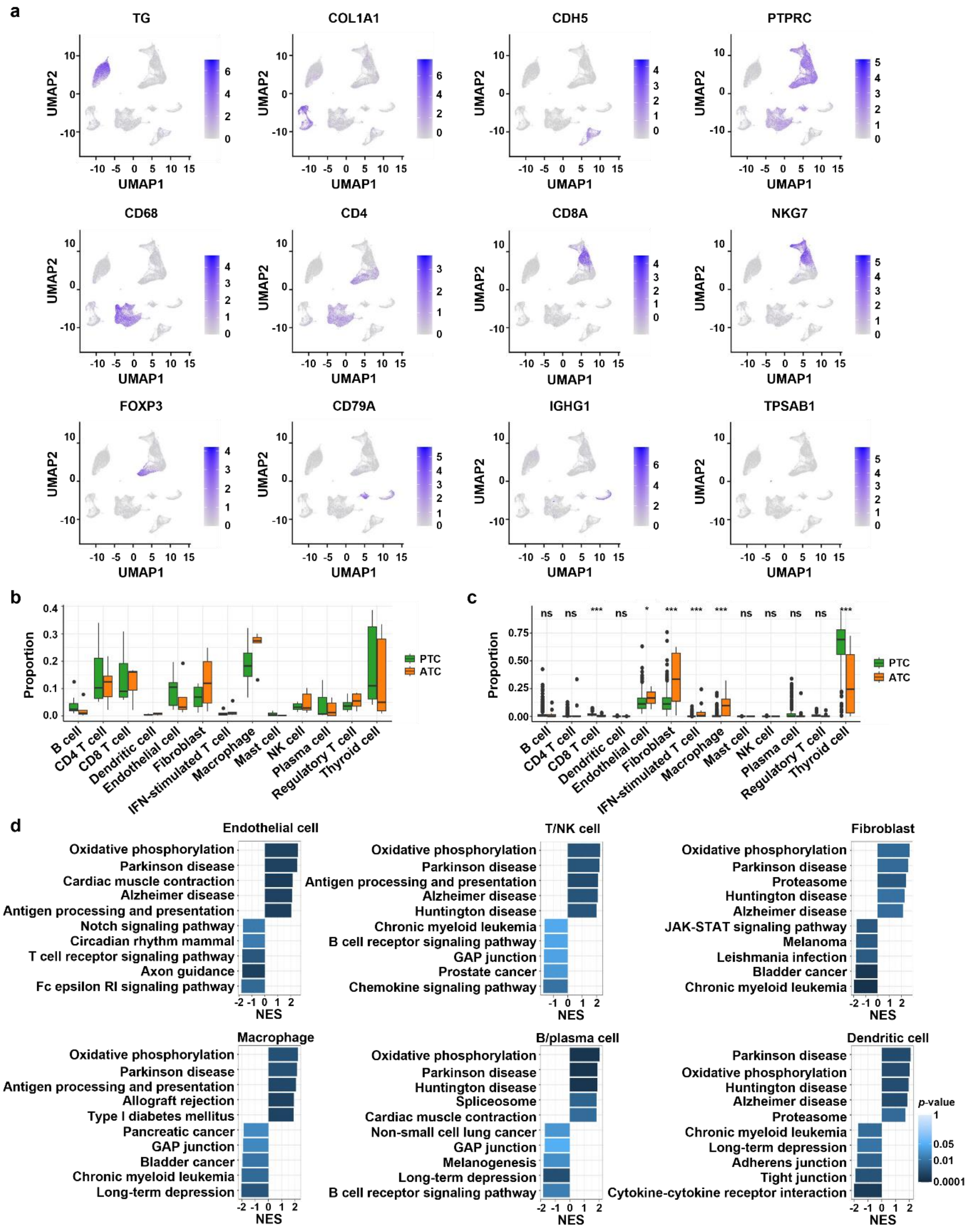

Supplementary Figure 3. Comparing tumor microenvironments between PTC and ATC. **a** UMAP plots showing the expression of cell type markers. **b, c** Bar plots comparing the proportion of cell types between PTC and ATC from single-cell RNA sequencing data (**b**) and from bulk RNA sequencing data (**c**) after deconvolution with CIBERSORTx. Data were expressed as the mean ± SD. A student's t test (two-sided) was used for statistical analysis. Exact p values can be found in source data file. (**d**) Differentially enriched KEGG pathways from GSEA between PTC and ATC within each cell type. P-value of gene sets enriched analysis (GSEA) was performed using the fgsea R package. PTC, papillary thyroid cancer; ATC, anaplastic thyroid cancer; NES, normalized enrichment score. *, p <0.05; ***, p <0.001; ns,

not significant. Source data are provided as a Source Data file.

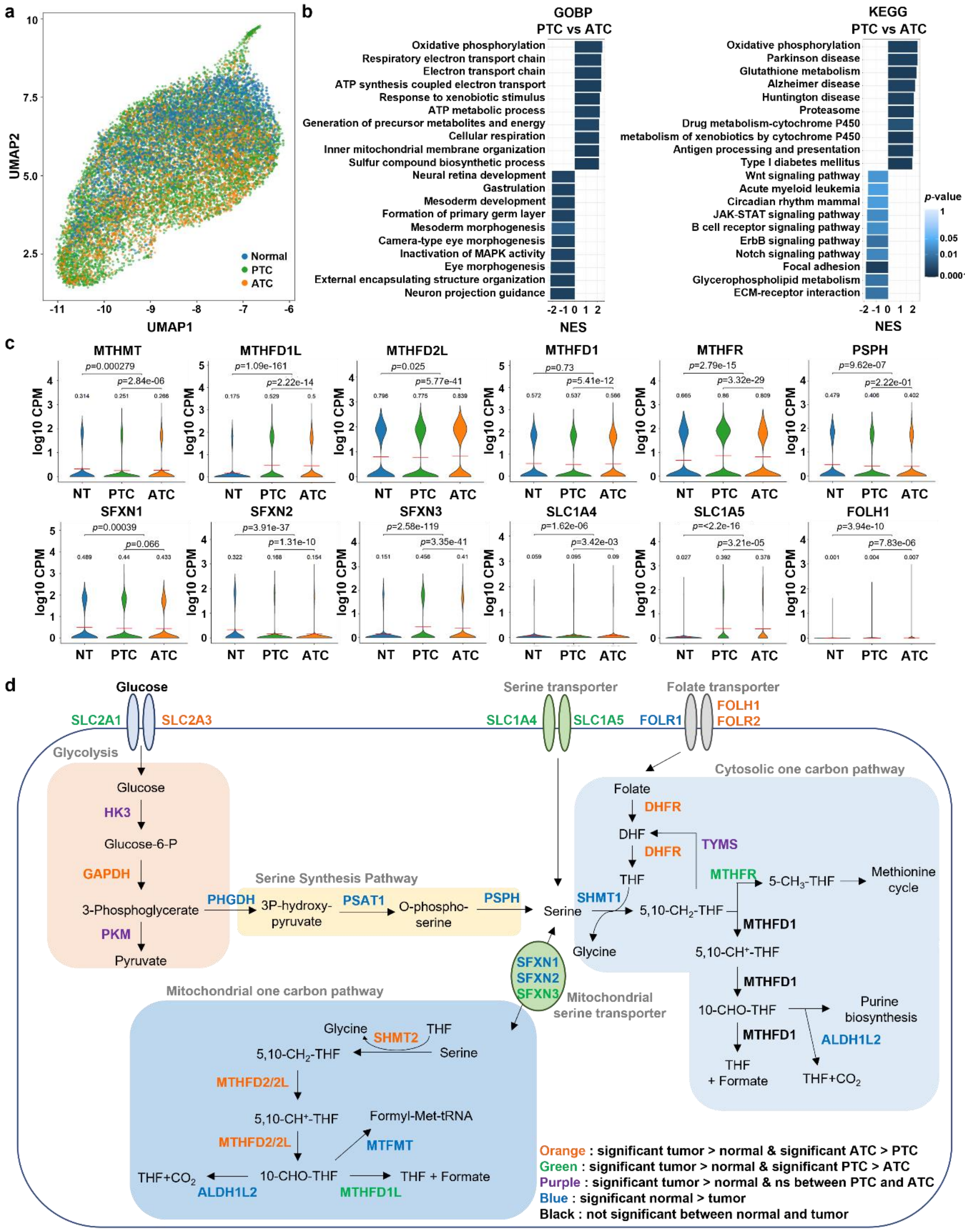

Supplementary Figure 4. Differentially expressed SGP genes among thyroid-origin cells in single-cell RNA sequencing data. **a** UMAP plot of all thyroid cells (n=17,412) colored by tissue types (PTC, n=10,633; ATC, n=3,575; normal thyroid follicular cells, n=3,204). Blue, normal thyroid cells; Green, PTC cells; Orange, ATC cells. **b** Differentially enriched GOBP (left) and KEGG (right) pathways from GSEA between tumor cells from PTC and ATC. *P*-value of gene sets enriched analysis (GSEA) was performed using the fgsea R package. **c** Violin plots comparing the expression levels of seringe/glycine metabolic pathway (SGP) genes

among normal thyroid, PTC, and ATC cells in single-cell RNA sequencing data. Data were expressed as the mean ± SD. A student's *t* test (two-sided) was used for statistical analysis. The involved pathways of the genes are illustrated in (**d**). Mean values are marked by red lines and are written at the top of each plot. **d** Illustration summarizing the expression of SGP genes among tissue types in each pathway. Colors of gene names indicate the difference among tissue types in single-cell RNA sequencing data. Orange, significantly induced in tumor compared to normal thyroid cells and significantly induced in ATC compared to PTC; Green, significantly induced in tumor compared to normal thyroid cells and significantly induced in PTC; Purple, significantly induced in tumor and not significant under comparison of PTC and ATC; Blue, significantly induced in normal compared to tumor; Black, not significant under comparison of normal and tumor. PTC, papillary thyroid cancer; ATC, anaplastic thyroid cancer; NES, normalized enrichment score; CPM, count per million. *, p <0.05; ***, p <0.001; ****, p <0.0001; ns, not significant. Source data are provided as a Source Data file.

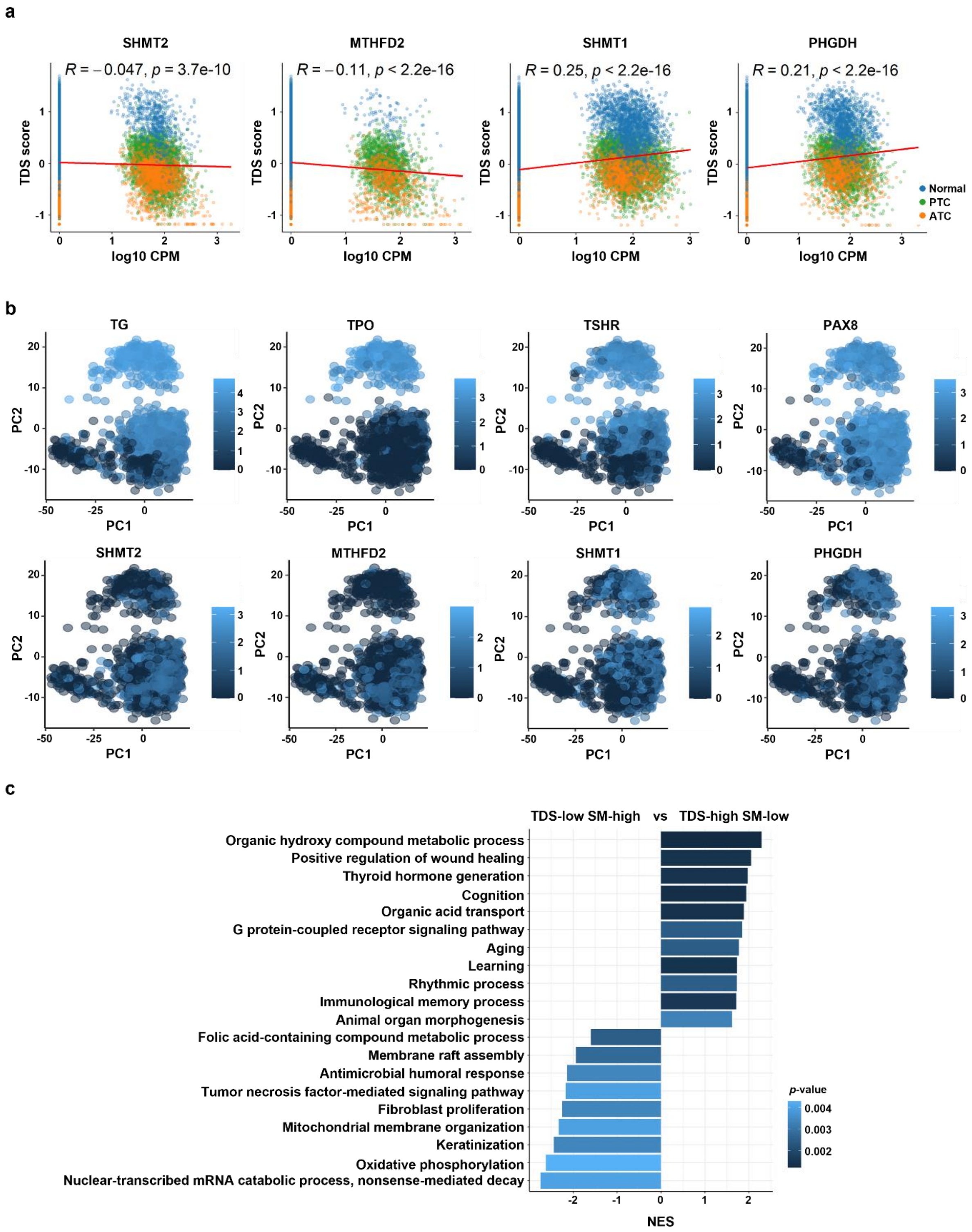

Supplementary Figure 5. Relationship between the mitochondrial one-carbon pathway and TDS in single-cell RNA sequencing data. a Scatter plots showing the correlation between expression levels (log10 CPM) of *SHMT2*, *MTHFD2*, *SHMT1*, or *PHGDH* and thyroid differentiation score (TDS). Pearson's correlation coefficients (Rs) and *p* values (two-side) are annotated at the top of each plot. Dots represent single cells colored by tissue types, and red lines indicate the fitted linear regression. **b** PCA plots showing expression of TDS (*TG*, *TPO*, *TSHR*, and *PAX8)* and SGP genes (*SHMT2*, *MTHFD2*, *SHMT2*, and *PHGDH*). **c** Differentially enriched GOBP pathways from GSEA between TDS-high SM-low and TDS-low SM-high. SM, mean of *SHMT2* and *MTHFD2* expression levels. *P*-value of gene sets enriched analysis

(GSEA) was performed using the fgsea R package. CPM, count per million; SM, mean of *SHMT2* and *MTHFD2* expression levels; NES, normalized enrichment score. Source data are provided as a Source Data file.

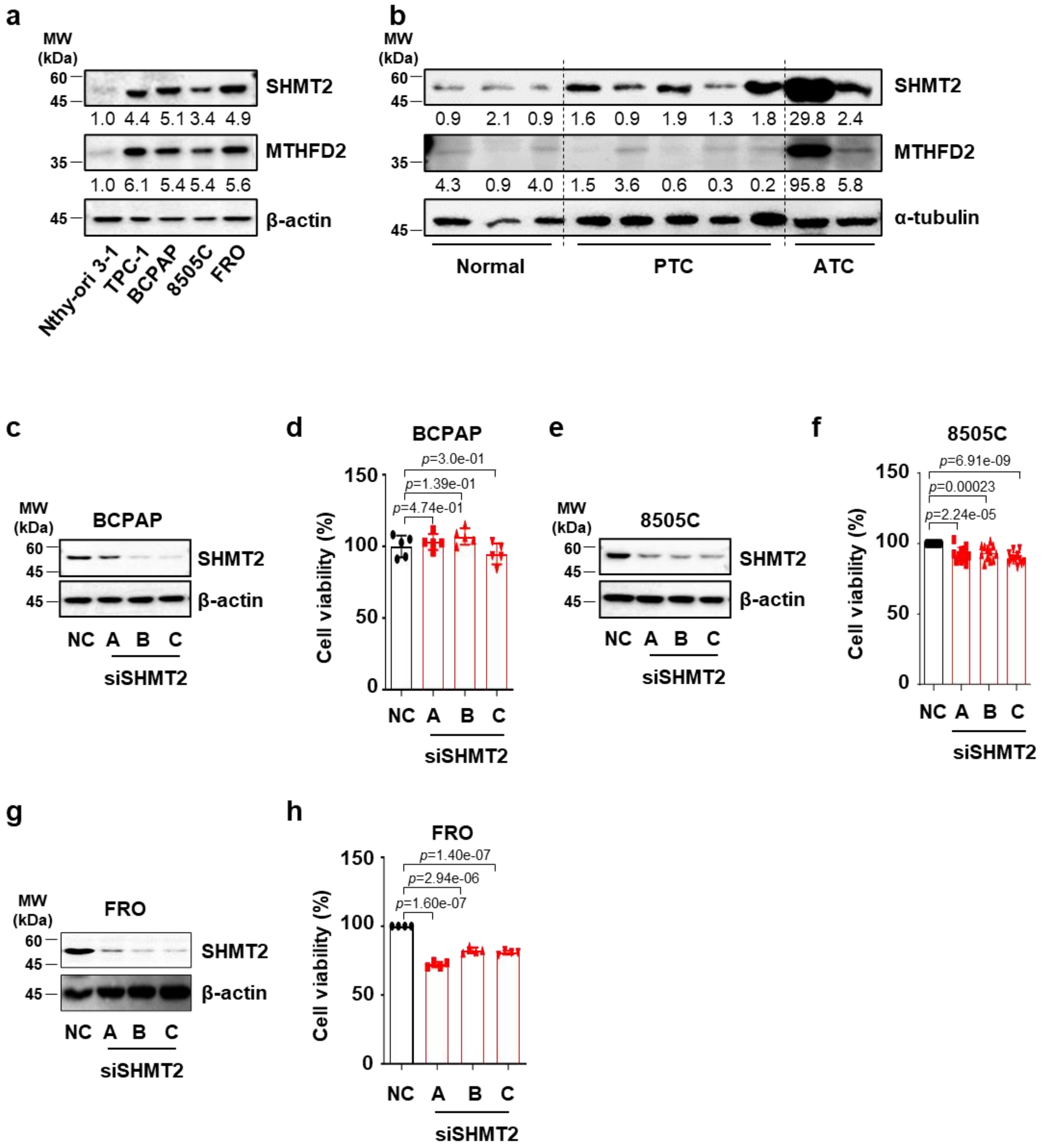

Supplementary Figure 6. Down-regulation of *SHMT2* by siRNA reduced cell viability and mitochondrial respiration of thyroid cancer cells. a Western blot images showing protein levels of SHMT2 and MTHFD2 in thyroid cancer cell lines. TPC-1 and BCPAP cells were differentiated PTC cell lines. 8505C and FRO cells were ATC cell lines. **b** Western blot images showing protein levels of SHMT2 and MTHFD2 in normal, PTC, and ATC tissues. **c, d** Western blot image (**c**) and bar plot (**d**) showing SHMT2 expression (**c**) and cell viability (**d**) from three *SHMT2* knock-down experiments using 20 nM siRNA in BCPAP cells for 48 hours (n=5 per group from more than three independent experiment). **e, f** Western blot image (**e**) and bar plot (**f**) showing SHMT2 expression (**e**) and cell viability (**f**) from three *SHMT2* knock-down experiments using 20 nM siRNA in 8505C cells for 48 hours (n=12 per group from three

independent experiment). **g, h** Western blot image (**g**) and bar plot (**h**) showing SHMT2 expression (**g**) and cell viability (**h**) from three *SHMT2* knock-down experiments using 20 nM siRNA in FRO cells for 48 hours (n=4 per group from more than three independent experiment). PTC, papillary thyroid cancer; ATC, anaplastic thyroid cancer. All experiments are representative of more than three independent experiments. Data were expressed as the mean ± SD. A student's *t* test (two-sided) was used for statistical analysis. Source data are provided as a Source Data file.

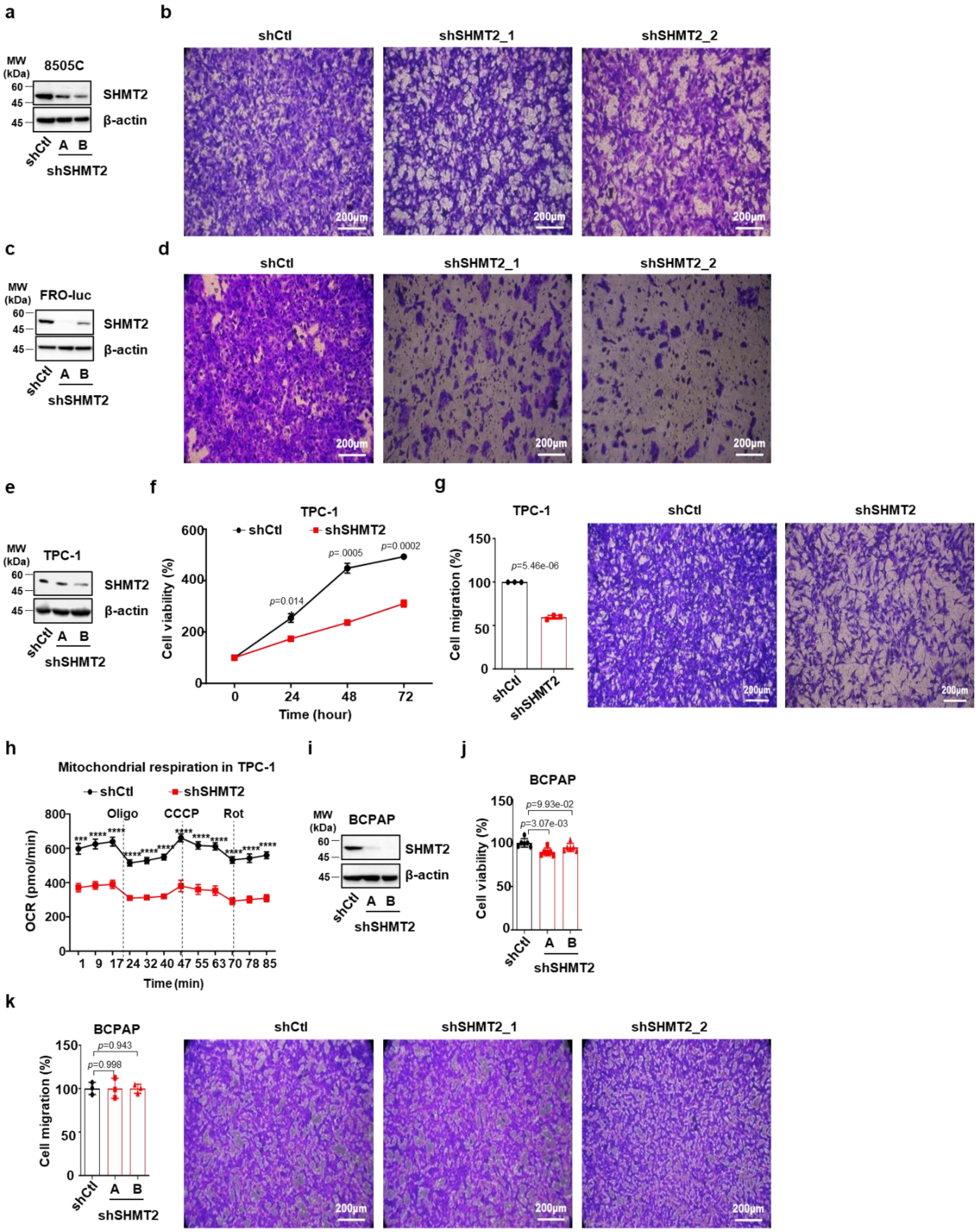

Supplementary Figure 7. Down-regulated *SHMT2* via shRNA lentiviral infection reduced cell viability, migration, and mitochondrial respiration in thyroid cancer cells. a Western blot image showing SHMT2 expression in shControl- and shSHMT2-8505C cells. **b** Microscopic images of stained cells comparing cell migration between shControl- and shSHMT2-8505C cells. **c** Western blot image showing SHMT2 expression in shControl- and shSHMT2-FRO cells. **d** Microscopic images of stained cells comparing cell migration between shControl- and shSHMT2-FRO cells. **e** Western blot image showing reduced SHMT2 expression by *SHMT2* knock-down in TPC-1 cells. **f** Line plot showing the decrease of cell viability in shSHMT2-TPC-1 cells (red square) compared to shControl-TPC-1 cells (black circle). For (f), n=3 per group from biological replicates. **g** Microscopic images and bar plot comparing cell migration between the shSHMT2-TPC-1 cells and shControl-TPC-1 cells (n=3 per group from biological replicates). **h** Line plot showing decreased OCR in shSHMT2-TPC-1 cells (red square) compared to shControl cells (black circle). For OCR, n=6 biologically independent samples per group. Exact *p* values can be found in source data file. **i** Western blot image showing the expression of SHMT2 in shControl- and shSHMT2-BCPAP cells. **j** Bar plot comparing cell viability between shSHMT2- and shControl-BCPAP cells (n=6 biologically independent samples per group). **k** Microscopic images and bar plot comparing cell migration in shSHMT2-BCPAP cells compared to shControl-BCPAP cells (n=3 biologically independent samples per group). All results are representative of more than three independent experiments. All experiments are representative of more than three independent experiments. Data were expressed as the mean ± SD. A student's *t* test (two-sided) was used for statistical analysis. ***, p<0.001; ****, p <0.0001. Source data are provided as a Source Data file.

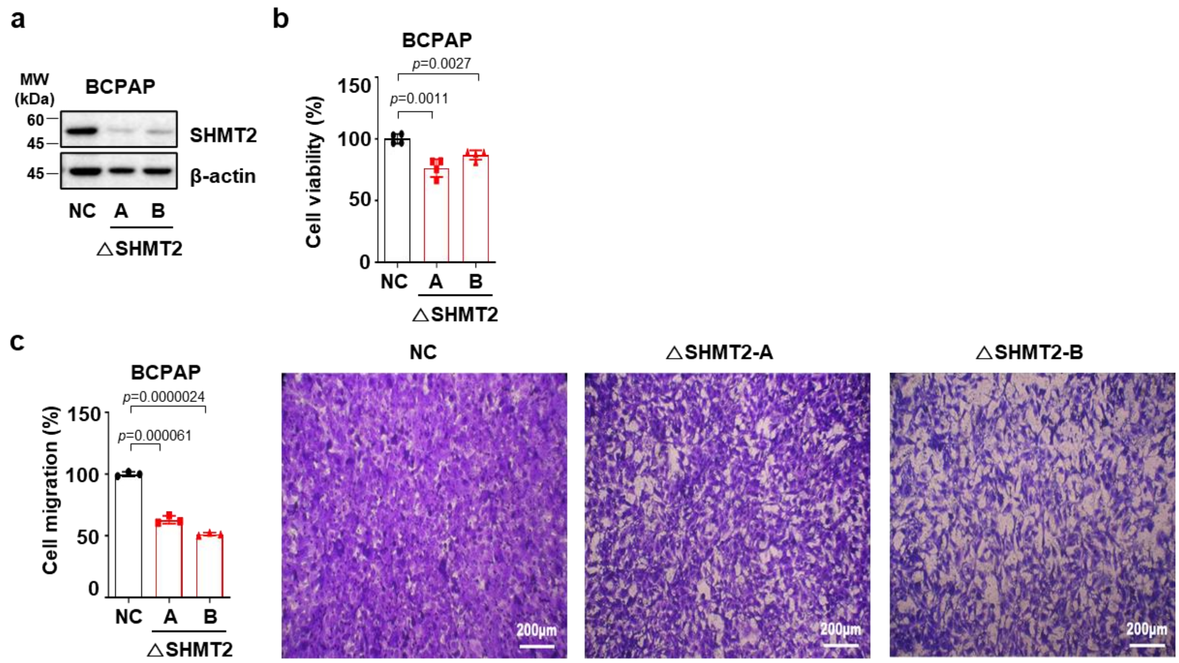

Supplementary Figure 8. Down-regulation of *SHMT2* using CRISPR/Cas9 system reduced cell viability and migration in PTC cell line.

a Western blot image showing the expression of sgSHMT2-BCPAP and control BCPAP cells. **b** Bar plot comparing cell viability between sgSHMT2-BCPAP and control BCPAP cells (n=4 biologically independent samples per group). **c** Microscopic images and bar plots comparing cell migration in sgSHMT2-BCPAP cells compared to control-BCPAP cells. Error bars indicate standard deviations from n=3 biological replicates. All experiments are representative of more than three independent experiments. Data were expressed as the mean ± SD. A student's *t* test

(two-sided) was used for statistical analysis. △SHMT2, reduced expression by single-guide RNA sequence targeting *SHMT2* using the CRISPR/Cas9 system; NC, negative control. Source data are provided as a Source Data file.

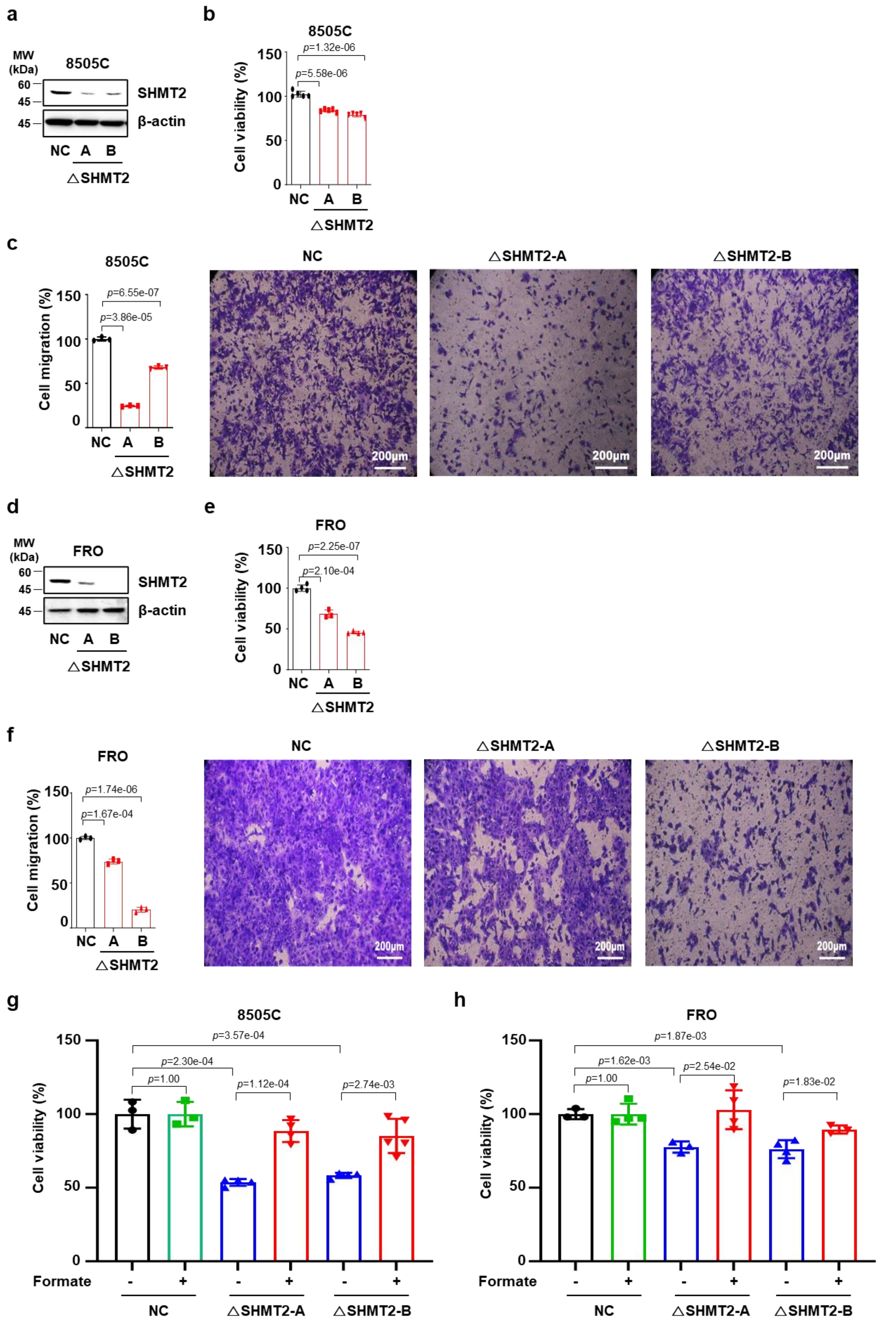

Supplementary Figure 9. Down-regulation of *SHMT2* using CRISPR/Cas9 system reduced cell viability and migration in ATC cell line.

a Western blot showing the expression of sgSHMT2- and control-8505C cells. **b** Bar plot comparing cell viability between sgSHMT2- and control-8505C cells (n=5 biological replicates). **c** Microscopic images and bar plots comparing cell migration in sgSHMT2- and control-8505C cells. Error bars indicate standard deviations from n=3 biological replicates. **d** Western blot showing the expression of sgSHMT2- and control-FRO cells. **e** Bar plot comparing cell viability between sgSHMT2- and control-FRO cells (n=4 biologically independent samples per group). **f** Microscopic images and bar plot comparing cell migration in sgSHMT2-FRO cells compared to control-FRO cells (n=3 biologically independent samples per group). **g** Bar plot showing viability of sgSHMT2-8505C cells treated with or without formate. Error bars indicate standard deviations from n=4 biological replicates. **h** Bar plot showing the viability of sgSHMT2-FRO cells treated with or without formate. Error bars indicate standard deviations from n=4 biological replicates. All results are representative of at least three independent experiments. Data were expressed as the mean ± SD. A student's *t* test (two-sided) was used for statistical analysis. △SHMT2, reduced expression by single-guide RNA sequence targeting *SHMT2* using the CRISPR/Cas9 system NC, negative control. Source data are provided as a Source Data file.

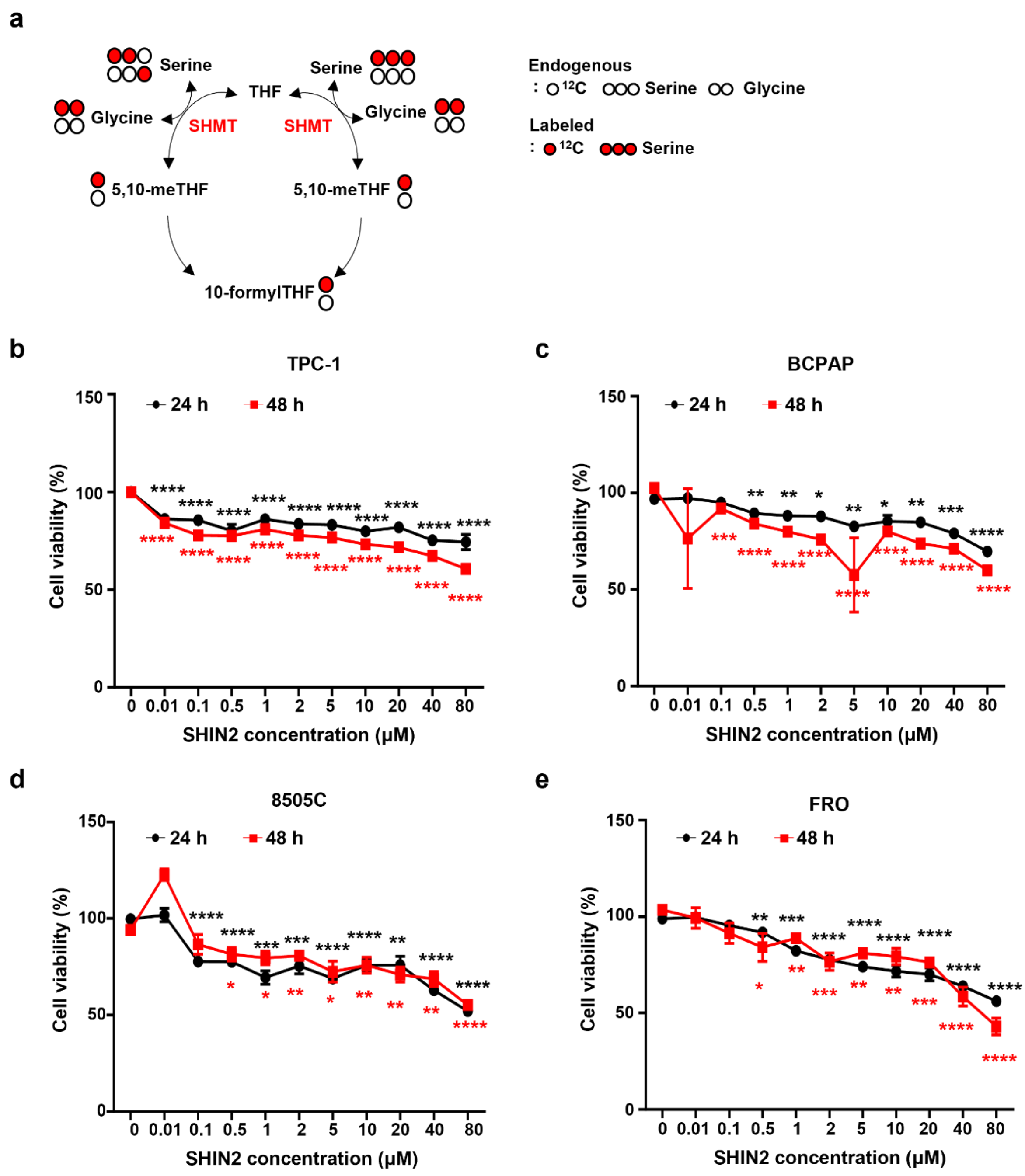

Supplementary Figure 10. SHIN2 reduced cell viability in thyroid cancer cells. a Schematic representation of the serine-derived carbon fates by SHIN2 treatment (20 µM, 24 h) using [U-^{13}C serine]. **b-e** Line plots showing cell viability by various concentrations of SHIN2 for 24 h (black) or 48 h (red) in TPC-1, BCPAP, 8505C, and FRO cells (n=4 biologically independent samples per group). All results are representative of at least three independent experiments. Data were expressed as the mean ± SD. A student's *t* test (two-sided) was used for statistical analysis. *, p<0.05; **, p <0.01; ***, p <0.001; ****, p>0.0001. Exact p values shown in b-e can be found in source data file. Source data are provided as a Source Data file.

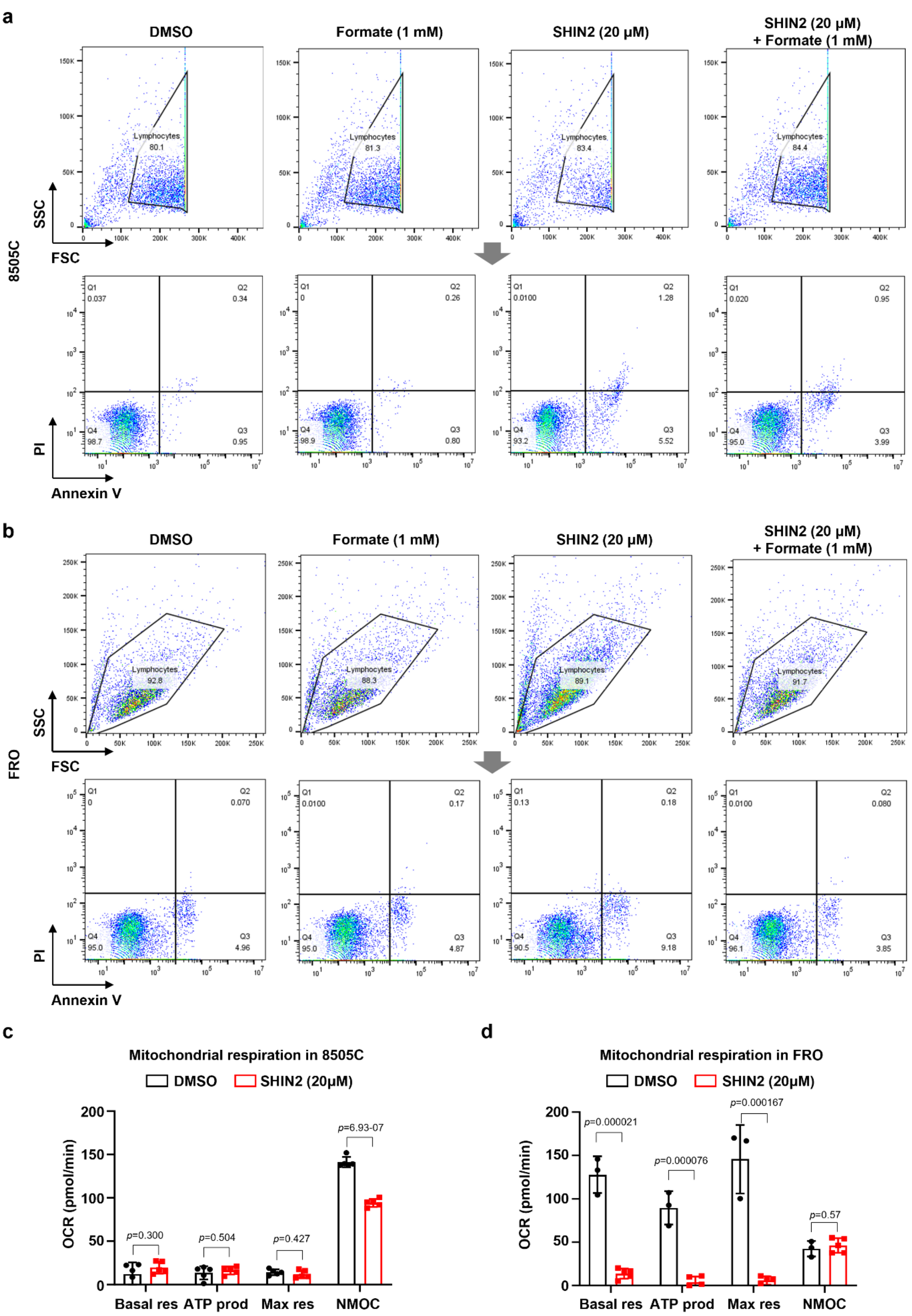

Supplementary Figure 11. SHIN2 reduced apoptotic cell death and mitochondrial respiration in undifferentiated thyroid cancer cells. a, b Scatter plots showing apoptosis

analysis using the Annexin V-FITC/PI assay after 20 μM SHIN2 with or without 1mM formate for 24 h in 8505C (**a**) and FRO (**b**) cells (n=3 biologically independent samples per group). **c, d** Bar plots showing OCR after 20 μM SHIN2 treatment for 24 h in 8505C (**c**) and FRO (**d**) cells (n=5 biologically independent samples per group). Basal respiration (Basal res), ATP production (ATP prod), maximal respiration (Max res), and non-mitochondrial respiration (NMOC) were used as respiratory parameters. Black, DMSO; Red, SHIN2 treatment. PI, propidium iodide; OCR, oxygen consumption rate. All results were representative of more than three independent experiments. Data were expressed as the mean ± SD. A student's *t* test (two-sided) was used for statistical analysis. Source data are provided as a Source Data file.

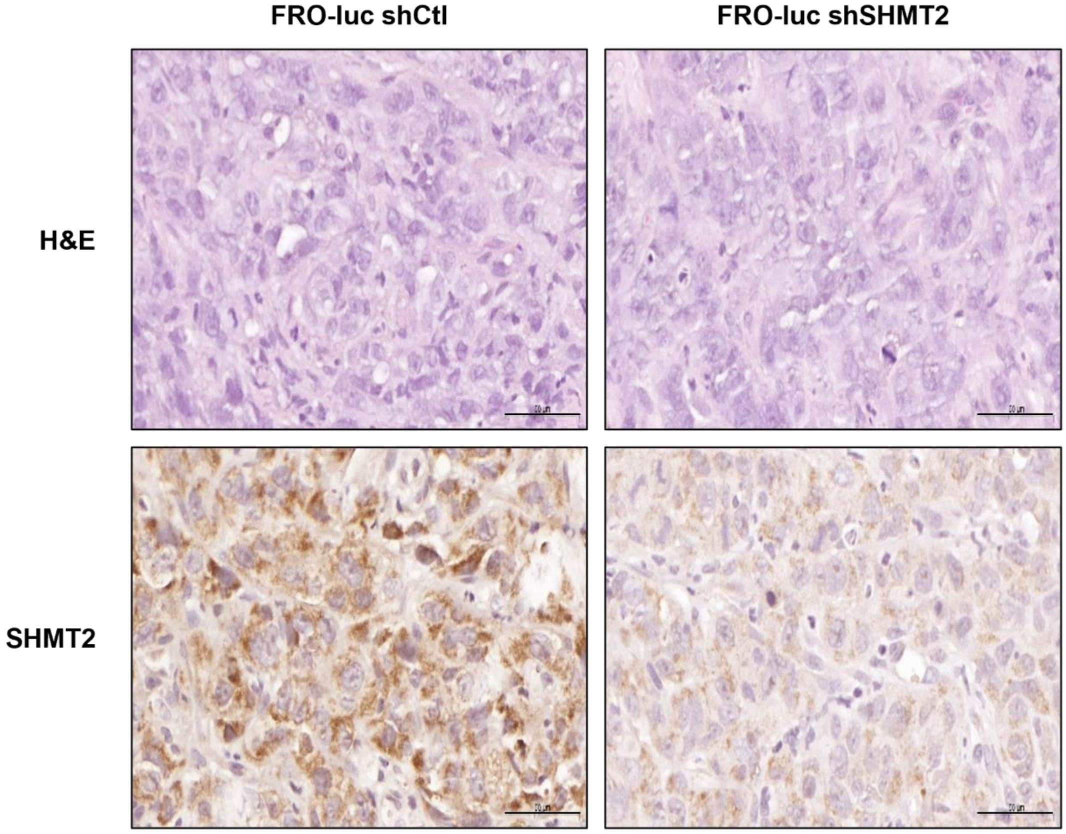

Supplementary Figure 12. Expression of SHMT2 reduced in mice injected with shSHMT2-FRO-luc cells. Representative imuunohistochemically stained images of SHMT2 in shSHMT2- or shControl-FRO-luc cell-injected mice (scale bar = 50 μm). H&E, hematoxylin and eosin; FRO-luc, luciferase-expressing FRO. Thease experiments were performed independently at least three times.

Supplementary Table 1. Relationship between SHMT2 expression and clinicopathologic factors in patients with thyroid cancer (N=369)

Variable		Number of patients	*SHMT2* expression		
			Low (N=184)	High (N=185)	*p*-value
Age, years			50.2±14.6	51.6±16.4	0.365
Sex	Male	88	46 (25.0)	42 (22.7)	0.605
	Female	281	138 (75.0)	143 (77.3)	
Tumor size, cm			1.50±1.43	2.15±2.07	0.001
Extracapsular invasion	No	157	90 (48.9)	67 (36.2)	0.014
	Yes	212	94 (51.1)	118 (63.8)	
Extrathyroidal extension	No	198	97 (52.7)	101 (54.6)	0.718
	Yes	171	87 (47.3)	84 (45.4)	
Central lymphnodemeta	No	171	93 (50.5)	78 (42.2)	0.106
	Yes	198	91 (49.5)	107 (57.8)	
Lateral lymphnodemeta	No	237	125 (67.9)	112 (60.5)	0.138
	Yes	132	59 (32.1)	73 (39.5)	
Lymphovascular invasion	No	58	33 (17.9)	25 (13.5)	0.243
	Yes	311	151 (82.1)	160 (86.5)	
Recurrence	No	316	161 (87.5)	155 (83.8)	0.309
	Yes	53	23 (12.5)	30 (16.2)	
Distant metastasis	No	347	178 (96.7)	169 (91.4)	0.029
	Yes	22	6 (3.3)	16 (8.6)	
Cancer type	PTC primary	348	181 (98.4)	167 (90.3)	0.003
	PDTC primary	5	1 (0.5)	4 (2.2)	
	ATC primary	16	2 (1.1)	14 (7.6)	

Data are presented as n (%) or mean ± standard deviation unless otherwise noted. P-values were obtained from unpaired t-tests (two-sided) for continuous parametric variables and the Mann–Whitney U test (two-sided) for nonparametric variables. The chi-squared test (two-sided) and Fisher's exact test (two-sided) were used to evaluate the significance of the correlations of SHMT2 expression with clinical and pathological parameters. AJCC, American Joint Committee on Cancer; SHMT2, serine hydroxymethyltransferase 2.

Supplementary Table 2. Relationship between MTHFD2 expression and clinicopathological factors in patients with thyroid cancer (N=369)

Variable		Number of patients	*MTHFD2* expression		
			Low (N=184)	High (N=185)	*p*-value
Age, years			50.6±15.0	51.2±16.0	0.692
Sex	Male	88	44 (23.9)	44 (23.8)	0.977
	Female	281	140 (76.1)	141 (76.2)	
Tumor size, cm			1.62±1.52	2.04±2.04	0.028
Extracapsular invasion	No	157	86 (46.7)	71 (38.4)	0.104
	Yes	212	98 (53.3)	114 (61.6)	
Extrathyroidal extension	No	198	96 (52.2)	102 (55.1)	0.568
	Yes	171	88 (47.8)	83 (44.9)	
Central lymphnodemeta	No	171	89 (48.4)	82 (44.3)	0.436
	Yes	198	95 (51.6)	103 (55.7)	
Lateral lymphnodemeta	No	237	119 (64.7)	118 (63.8)	0.858
	Yes	132	65 (35.3)	67 (36.2)	
Lymphovascular invasion	No	58	30 (16.3)	28 (15.1)	0.758
	Yes	311	154 (83.7)	157 (84.9)	
Recurrence	No	316	160 (87.0)	156 (84.3)	0.471
	Yes	53	24 (13.0)	29 (15.7)	
Distant metastasis	No	347	179 (97.3)	168 (90.8)	0.009
	Yes	22	5 (2.7)	17 (9.2)	
Cancer type	PTC primary	348	181 (98.4)	167 (90.3)	0.001
	PDTC primary	5	2 (1.1)	3 (1.6)	
	ATC primary	16	1 (0.5)	15 (8.1)	

Data are presented as n (%) or mean ± standard deviation unless otherwise noted. P-values were obtained from unpaired t-tests (two-sided) for continuous parametric variables and the Mann–Whitney U test (two-sided) for nonparametric variables. The chi-squared test (two-sided) and Fisher's exact test (two-sided) were used to evaluate the significance of the correlations of MTHFD2 expression with clinical and pathological parameters. AJCC, American Joint Committee on Cancer; MTHFD2, methylenetetrahydrofolate dehydrogenase.

Supplementary Table 3. Relationship between TDS score and clinicopathological factors in patients with thyroid cancer (N=369)

Variable		Number of patients	TDS score		
			TDS < 0 (N=231)	TDS > 0 (N=138)	*p*-value
Age, years			51.6±15.6	49.7±15.4	0.238
Sex	Male	88	51 (22.1)	37 (26.8)	0.302
	Female	281	180 (77.9)	101 (73.2)	
Tumor size, cm			1.86±1.74	1.77±1.92	0.637
Extracapsular invasion	No	157	89 (38.5)	68 (49.3)	0.043
	Yes	212	142 (61.5)	70 (50.7)	
Extrathyroidal extension	No	198	128 (55.4)	70 (50.7)	0.382
	Yes	171	103 (44.6)	68 (49.3)	
Central lymphnodemeta	No	171	99 (42.9)	72 (52.5)	0.082
	Yes	198	132 (57.1)	66 (47.8)	
Lateral lymphnodemeta	No	237	141 (61.0)	96 (69.6)	0.098
	Yes	132	90 (39.0)	42 (30.4)	
Lymphovascular invasion	No	58	31 (13.4)	27 (19.6)	0.117
	Yes	311	200 (86.6)	111 (80.4)	
Recurrence	No	316	200 (86.6)	116 (84.1)	0.504
	Yes	53	31 (13.4)	22 (15.9)	
Distant metastasis	No	347	214 (92.6)	133 (96.4)	0.142
	Yes	22	17 (7.4)	5 (3.6)	
Cancer type	PTC primary	348	215 (93.1)	133 (96.4)	0.005
	PDTC primary	5	1 (0.4)	4 (2.9)	
	ATC primary	16	15 (6.5)	1 (0.7)	

Data are presented as n (%) or mean ± standard deviation unless otherwise noted. P-values were obtained from unpaired t-tests (two-sided) for continuous parametric variables and the Mann–Whitney U test (two-sided) for nonparametric variables. The chi-squared test (two-sided) and Fisher's exact test (two-sided) were used to evaluate the significance of the correlations of TDS with clinical and pathological parameters. TNM classification from the AJCC seventh edition was used. AJCC, American Joint Committee on Cancer; TDS, thyroid differentiation score.

Supplementary Table 4. Relationship between SHMT2 expression and clinicopathological factors in thyroid cancer patients in TCGA-THCA cohort (N=500)

Variable		Number of patients	*SHMT2* expression		
			Low (N=250)	High (N=250)	*p*-value
Age, years			45.5±15.5	49.0±16.0	0.015
Sex	Male	135	68 (27.2)	67 (26.8)	0.076
	Female	365	182 (72.8)	183 (73.2)	
Tumor size, cm			1.35±1.07	1.44±1.07	0.369
T stage	T1-T2	308	170 (68.0)	138 (55.2)	0.003
	T3-T4	192	80 (32.0)	112 (44.8)	
Extrathyroidal extension	No	482	248 (99.2)	234 (93.6)	0.001
	Yes	18	2 (0.8)	16 (6.4)	
Lymph node metastasis	No	277	152 (60.8)	125 (50.0)	0.015
	Yes	223	98 (39.2)	125 (50.0)	
M stage	M0	492	248 (99.2)	244 (97.6)	0.154
	M1	8	2 (0.8)	6 (2.4)	
Stage	I	284	157 (62.8)	127 (50.8)	0.000
	II	52	32 (12.8)	20 (8.0)	
	III	111	47 (18.8)	64 (25.6)	
	IV	53	14 (5.6)	39 (15.6)	
Overall survival	Alive	484	243 (97.2)	241 (96.4)	0.611
	Dead	16	7 (2.8)	9 (3.6)	
Disease-specific survival	Alive	493	248 (99.2)	245 (98.0)	0.253
	Dead	7	2 (0.8)	5 (2.0)	
Disease-free interval event	No	474	239 (95.6)	235 (94.0)	0.420
	Yes	26	11 (4.4)	15 (6.0)	
$BRAF^{V600E}$ mutation	No	268	150 (60.0)	118 (47.2)	0.004
	Yes	232	100 (40.0)	132 (52.8)	

Data are presented as n (%) or mean ± standard deviation unless otherwise noted. P-values were obtained from unpaired t-tests (two-sided) for continuous parametric variables and the Mann–Whitney U test (two-sided) for nonparametric variables. The chi-squared test (two-sided) and Fisher's exact test (two-sided) were used to evaluate the significance of the correlations of SHMT2 expression with clinical and pathological parameters. TNM classification from the AJCC seventh edition was used. AJCC, American Joint Committee on Cancer; SHMT2, serine hydroxymethyltransferase 2.

Supplementary Table 5. Relationship between MTHFD2 expression and clinicopathological factors in thyroid cancer patients in TCGA-THCA cohort (N=500)

Variable		Number of patients	*MTHFD2* expression		
			Low (N=250)	High (N=250)	*p*-value
Age, years			47.8±15.2	46.8±16.5	0.462
Sex	Male	135	68 (27.2)	67 (26.8)	0.920
	Female	365	182 (72.8)	183 (73.2)	
Tumor size, cm			1.43±1.11	1.36±1.03	0.475
T stage	T1-T2	308	159 (63.6)	149 (59.6)	0.358
	T3-T4	192	91 (36.4)	101 (40.4)	
Extrathyroidal extension	No	482	243 (97.2)	239 (95.6)	0.337
	Yes	18	7 (2.8)	11 (4.4)	
Lymph node metastasis	No	277	168 (67.2)	109 (43.6)	0.000
	Yes	223	82 (32.8)	141 (56.4)	
M stage	M0	492	245 (98.0)	247 (98.8)	0.476
	M1	8	5 (2.0)	3 (1.2)	
Stage	I	284	137 (54.8)	147 (58.8)	0.024
	II	52	33 (13.2)	19 (7.6)	
	III	111	61 (24.4)	50 (20.0)	
	IV	53	19 (7.6)	34 (13.6)	
Overall survival	Alive	484	243 (97.2)	241 (96.4)	0.611
	Dead	16	7 (2.8)	9 (3.6)	
Disease-specific survival	Alive	493	247 (98.8)	246 (98.4)	0.703
	Dead	7	3 (1.2)	4 (1.6)	
Disease-free interval event	No	474	238 (95.2)	236 (94.4)	0.687
	Yes	26	12 (4.8)	14 (5.6)	
$BRAF^{V600E}$ mutation	No	268	159 (63.6)	109 (43.6)	0.000
	Yes	232	91 (36.4)	141 (56.4)	

Data are presented as n (%) or mean ± standard deviation unless otherwise noted. P-values were obtained from unpaired t-tests (two-sided) for continuous parametric variables and the Mann–Whitney U test (two-sided) for nonparametric variables. The chi-squared test (two-sided) and Fisher's exact test (two-sided) were used to evaluate the significance of the correlations of MTHFD2 expression with clinical and pathological parameters. TNM classification from the AJCC seventh edition was used. AJCC, American Joint Committee on Cancer; MTHFD2, methylenetetrahydrofolate dehydrogenase.
