# Supplementary material for: Unraveling the role of the mitochondrial one-carbon pathway in undifferentiated thyroid cancer by multi-omics analyses
Source: Nat Commun. 2024 Feb 8;15:1163. doi: 10.1038/s41467-024-45366-0 (PMC10853200; doi:10.1038/s41467-024-45366-0)
Supplement: Supplementary file 3 — Reporting Summary [file 41467_2024_45366_MOESM3_ESM.pdf]

nature portfolio

Corresponding author(s): Yea Eun Kang
Bon Seok Koo
Seon Kyu Kim

Last updated by author(s): Dec 5, 2023

Reporting Summary

Nature Portfolio wishes to improve the reproducibility of the work that we publish. This form provides structure for consistency and transparency in reporting. For further information on Nature Portfolio policies, see our Editorial Policies and the Editorial Policy Checklist.

Statistics

For all statistical analyses, confirm that the following items are present in the figure legend, table legend, main text, or Methods section.

n/a	Confirmed	
☐	☒	The exact sample size (n) for each experimental group/condition, given as a discrete number and unit of measurement
☐	☒	A statement on whether measurements were taken from distinct samples or whether the same sample was measured repeatedly
☐	☒	The statistical test(s) used AND whether they are one- or two-sided<br>*Only common tests should be described solely by name; describe more complex techniques in the Methods section.*
☐	☒	A description of all covariates tested
☐	☒	A description of any assumptions or corrections, such as tests of normality and adjustment for multiple comparisons
☐	☒	A full description of the statistical parameters including central tendency (e.g. means) or other basic estimates (e.g. regression coefficient) AND variation (e.g. standard deviation) or associated estimates of uncertainty (e.g. confidence intervals)
☐	☒	For null hypothesis testing, the test statistic (e.g. F, t, r) with confidence intervals, effect sizes, degrees of freedom and P value noted<br>*Give P values as exact values whenever suitable.*
☒	☐	For Bayesian analysis, information on the choice of priors and Markov chain Monte Carlo settings
☐	☒	For hierarchical and complex designs, identification of the appropriate level for tests and full reporting of outcomes
☐	☒	Estimates of effect sizes (e.g. Cohen's d, Pearson's r), indicating how they were calculated

Our web collection on statistics for biologists contains articles on many of the points above.

Software and code

Policy information about availability of computer code

Data collection	For metabolomics data acquisition, an Agilent 1260 HPLC system with an Agilent 6460 triple quadrupole (QqQ)-based mass spectrometer (MS) was used, and data extraction was performed using XCMS Online (http://xcmsonline.scripps.edu).<br><br>For bulk RNA sequencing, extracted RNA was used to contruct RNA libraries using the TruSeq RNA access library or a stranced mRNA LT sample prep kit (Illumina Inc.). All samples were sequenced on an Illumina HiSeq2500.<br><br>TCGA data were obtained from the TCGA Data Portal (http://tcga-data.nci.nih.gov).<br><br>For singcle-cell RNA sequencing, samples were sequenced using a HiSeq X (illumina).<br><br>To measure the oxygen consumption rate (OCR), we used XF24 analyzer (Agilent).<br><br>Flow cytometry analysis was performed using a BD LSRFortessa flow cytometer (BD Biosciences).<br><br>For imaging tumors in live animals, images were acquired with the bioluminescence imaging system (IVIS) consisting of a Lumina XRMS instrument (PerkinElmer).
Data analysis	Sequenced reads were mapped to the human reference genome (GRCh38). Custom codes were written by R (v4.3.0).Following packages were used in R : ggplot2(v3.4.2), corrplot(v0.92), ComplexHeatmap(v2.16.0), DESeq2 (v1.40.1), fgsea(v1.26.0), and Seurat(v4.3.0).

For metabolomics analysis, MassHunter Quantitative Analysis software (Agilent) was used for peak area integration.

In western blot, images were acquired using the Odyssey imaging system and quantified using Image Studio Digits (LI-COR Biosciences).

Flow cytometry data were analyzed using FlowJo software (FlowJo).

For statistical analysis, data were analysis using Prisml0 (GraphPad Software Inc.) and SPSS version 26 (IBM Corp.).

For manuscripts utilizing custom algorithms or software that are central to the research but not yet described in published literature, software must be made available to editors and reviewers. We strongly encourage code deposition in a community repository (e.g. GitHub). See the Nature Portfolio guidelines for submitting code & software for further information.

Data

Policy information about availability of data

All manuscripts must include a data availability statement. This statement should provide the following information, where applicable:

- Accession codes, unique identifiers, or web links for publicly available datasets
- A description of any restrictions on data availability
- For clinical datasets or third party data, please ensure that the statement adheres to our policy

The accession number for the bulk RNA sequencing dataset is GSE213647 and the accession numbers for single-cell RNA of thyroid cancer (PTC and ATC) are GSE232237.

Research involving human participants, their data, or biological material

Policy information about studies with human participants or human data. See also policy information about sex, gender (identity/presentation), and sexual orientation and race, ethnicity and racism.

Reporting on sex and gender	The information on sex is in the Supplementary tables. However, we did not perform any sex-based analysis.
Reporting on race, ethnicity, or other socially relevant groupings	All patients are Korean. And we did not analyze by on race, ethnicity, or other socially relevant grouping.
Population characteristics	For bulk RNA-seq, tissues were obtained from a total 632 patients, including 263 normal thyroid tissues and thyroid tissues from 348 PTC, 5 PTDC, and 16 ATC patients. For scRNA-seq, single cells were isolated from 5 normal thyroid tissues, 7 PTC tissues, and 5 ATC tissues.<br><br>The average age of PTC patients is 49.8±14.94, PD patients is 61.8±19.29, and ATC patients is 71.31±10.63.<br>We idendified no difference between cancer type and sex through chi-squre test (p=0.159).
Recruitment	All patients with thyroid cancer that was planed to undergo surgery could be asked to be asked to be recruited. So, informed consent was obtained from all participants.<br><br>We analyzed RNA sequencing after identifying that cancer type, sex, and age are not significant related.
Ethics oversight	This study was approved by the Institutional Research and Ethics Committee at Chungnam National University Hospital, Seoul National University Hospital, and Seoul National University Bundang Hospital.

Note that full information on the approval of the study protocol must also be provided in the manuscript.

Field-specific reporting

Please select the one below that is the best fit for your research. If you are not sure, read the appropriate sections before making your selection.

[x] Life sciences   [ ] Behavioural & social sciences   [ ] Ecological, evolutionary & environmental sciences

For a reference copy of the document with all sections, see nature.com/documents/nr-reporting-summary-flat.pdf

Life sciences study design

All studies must disclose on these points even when the disclosure is negative.

Sample size	No statistical methods were used to predetermine to sample size. The sample size for all other experiments was chosen to include at least 3 biologically independent experiments.
Data exclusions	No data bas been excluded.
Replication	All in vitro and in vivo experiments were performed at least three times independent experiment, and we confirmed reproducibility of the experimental finding.

Randomization	Human tissue samples were allocated to groups based on disease status (normal and tumor) if applicable.<br>In in vitro studies, cells were allocated into control group and drug-treated group randomly.<br>In in vivo studies, BALB/c nude mice were allocated into treatment group and control group randomly.
Blinding	Blinding was not applicable to this study since have no elements that might be affected by bias from the subject or observer.

Reporting for specific materials, systems and methods

We require information from authors about some types of materials, experimental systems and methods used in many studies. Here, indicate whether each material, system or method listed is relevant to your study. If you are not sure if a list item applies to your research, read the appropriate section before selecting a response.

Materials & experimental systems

n/a	Involved in the study
☐	☒ Antibodies
☐	☒ Eukaryotic cell lines
☒	☐ Palaeontology and archaeology
☐	☒ Animals and other organisms
☒	☐ Clinical data
☒	☐ Dual use research of concern
☒	☐ Plants

Methods

n/a	Involved in the study
☒	☐ ChIP-seq
☒	☐ Flow cytometry
☒	☐ MRI-based neuroimaging

Antibodies

Antibodies used	1) Rabbit polyclonal anti-SHMT2 : Cell signaling, Cat# 12762 (1:1000 for immunoblots)<br>2) Rabbit polyclonal anti-MTHFD2 : abcam, Cat# ab151447 (1:1000 for immunoblots)<br>3) Rabbit polyclonal beta-actin : abcam, Cat# ab8227 (1:1000 for immunoblots)<br>4) Mouse monoclonal anti-alpha-tubulin : Sigma Aldrich, Cat# T5168(1:1000 for immunoblots)<br>5) Mouse monoclonal Total OXPHOS Rodent antibody cocktail : abcam, Cat# ab110413 (1:1000 for immunoblots)<br>6) Goat anti-rabbit IgG-HRP : Santa cruz, Cat# sc-2030 (1:2000 for immunoblots)<br>7) Goat anti-mouse IgG-HRP : Santa cruz , Cat# sc-2005 (1:1000 for immunoblots)<br>8) Rabbit polyclonal anti-SHMT2 : Invitrogen, Cat# PA5-32228 (1:250 for immunohistochemistry)
Validation	All the antibodies were validated for the species (human) and applications (immunoblots, immunohistochemistry) by the correspondent manufacturer, which is described in the manufacturer's website. Our usage was described in the Materials and Methods section of the manuscript.

Eukaryotic cell lines

Policy information about cell lines and Sex and Gender in Research

Cell line source(s)	BCPAP (cat# ACC273) and 8505C (cat# ACC219) cells were purchased from DSMZ (Germany). Nthy-ori 3-1, TPC-1, FRO and luciferase-expressing FRO (FRO-Luc) cells were provided by Dr. Young Joo Park and Dr. Sun Wook Cho (Seoul National University College of Medicine, Seoul, Republic of Korea).
Authentication	BCPAP and 8505C cell lines were not authenticated since they were purchased commercially and not commonly misidentified.<br><br>Nthy-ori 3-1, TPC-1, and FRO were authenticated by the short tandem repeat typing method using AmplFLSTR identifiler PCR Amplification kit (cat.4322288, Applied Biosystems, Foster, CA, USA), 3530xL DNA Analyzer (Applied Biosystems), and GeneMapper v5 (Applied Biosystems) on May, 2020.
Mycoplasma contamination	All cell lines used in this study were tested negative for mycoplasma contamination.
Commonly misidentified lines<br>(See ICLAC register)	No commonly misidentified cell lines were used.

Animals and other research organisms

Policy information about studies involving animals; ARRIVE guidelines recommended for reporting animal research, and Sex and Gender in Research

Laboratory animals	The 6-week-old male BALB/c nude mice fed standard chow (Teklad 2018) were housed in a specific pathogen-free animal facility (Chungnam National University Hospital Preclinical Research Center) in a controlled environment (12 h light/12 h dark cycle; humidity, 50-60%; ambient temperature, 23℃.
Wild animals	Wild animals were not used.
Reporting on sex	All in vivo experiments were conducted on male mice to avoid any effects of estrogen in thyroid cancer progression.
Field-collected samples	No field-collected samples were used.
Ethics oversight	All animal procedures- were performed according to the guidelines of the Institutional Animal Care at Chungnam National University.

Note that full information on the approval of the study protocol must also be provided in the manuscript.

Plants

Seed stocks	*Report on the source of all seed stocks or other plant material used. If applicable, state the seed stock centre and catalogue number. If plant specimens were collected from the field, describe the collection location, date and sampling procedures.*
Novel plant genotypes	*Describe the methods by which all novel plant genotypes were produced. This includes those generated by transgenic approaches, gene editing, chemical/radiation-based mutagenesis and hybridization. For transgenic lines, describe the transformation method, the number of independent lines analyzed and the generation upon which experiments were performed. For gene-edited lines, describe the editor used, the endogenous sequence targeted for editing, the targeting guide RNA sequence (if applicable) and how the editor was applied.*
Authentication	*Describe any authentication procedures for each seed stock used or novel genotype generated. Describe any experiments used to assess the effect of a mutation and, where applicable, how potential secondary effects (e.g. second site T-DNA insertions, mosiacism, off-target gene editing) were examined.*
