# Supplementary material for: Unraveling the role of the mitochondrial one-carbon pathway in undifferentiated thyroid cancer by multi-omics analyses
Source: Nat Commun. 2024 Feb 8;15:1163. doi: 10.1038/s41467-024-45366-0 (PMC10853200; doi:10.1038/s41467-024-45366-0)
Supplement: Supplementary file 4 — Description of Additional Supplementary Files [file 41467_2024_45366_MOESM4_ESM.docx]

**Description of Additional Supplementary Files**

File Name : Supplementary data 1

Description : Raw mass spectrometry data of metabolomics from papillary thyroid cancer (PTC) tumor tissues and paired normal tissues.

File Name : Supplementary data 2

Description : Differential expressed metabolites in PTC tumor tissues compared to paired normal tissues.

File Name : Supplementary data 3

Description : Differential expressed genes in anaplastic thyroid cancer (ATC) compared to PTC tumor tissues.

File Name : Supplementary data 4

Description : KEGG pathway of Gene-set enrichment analysis (GSEA) between ATC versus PTC.

File Name : Supplementary data 5

Description : Different-expressed genes in poorly differentiated thyroid cancer (PDTC) compared to PTC.

File Name : Supplementary data 6

Description : Enriched KEGG pathway comparison of between PDTC and PTC.

File Name : Supplementary data 7

Description : Differential expressed genes comparison of ATC versus PDTC.

File Name : Supplementary data 8

Description : Comparison of KEGG pathway in ATC compared to PDTC.

File Name : Supplementary data 9

Description : KEGG pathway of GSEA results in tumor compared to normal tissues.

File Name : Supplementary data 10

Description : Different-expressed metabolic genes in tumor compared to normal tissues.

File Name : Supplementary data 11

Description : Enriched KEGG pathway from GSEA between TDS-high and -low tumors.

File Name : Supplementary data 12

Description : Differential expressed metabolic genes in TDS-high compared to TDS-low tumors.

File Name : Supplementary data 13

Description : Sample information for RNA sequencing

File Name : Supplementary data 14

Description : Sample information for single-cell RNA sequencing

File Name : Supplementary data 15

Description : Short tandem repeat (STR) profiling results of cell lines
